# Auditory Processing Disorder Test Battery in European Portuguese—Development and Normative Data for Pediatric Population

**DOI:** 10.3390/audiolres11030044

**Published:** 2021-09-17

**Authors:** Jorge Humberto Martins, Marisa Alves, Susana Andrade, Isabel Falé, António Teixeira

**Affiliations:** 1 Serviço de Otorrinolaringologia, Centro Hospitalar Universitário de Coimbra, 3000-075 Coimbra, Portugal; marisalves2002@gmail.com (M.A.); Susanasousaandrade@gmail.com (S.A.); 2 Departamento de Humanidades, Universidade Aberta & Centro de Linguística da Universidade de Lisboa (CLUL), 1600-214 Lisboa, Portugal; ifale@campus.ul.pt; 3 Biomedical Informatics and Technologies (BIT), Institute of Electronics and Informatics Engineering of Aveiro (IEETA), Department of Electronics Telecommunications & Informatics, University of Aveiro, 3810-193 Aveiro, Portugal

**Keywords:** Auditory Processing assessment, Auditory Assessment Battery, auditory tests, children, normative data, verbal tests, non-verbal tests, dichotic tests, low redundancy tests, temporal processing tests

## Abstract

There is an increasing need for state-of-the-art Central Auditory Processing assessment for Portuguese native speakers, applicable as early as possible. As a contribution to answering this need, this paper presents a new battery for Central Auditory Processing assessment for European Portuguese applicable to children aged 5 and above, named BAPA-PE, providing information regarding test selection and development. The battery consists of six behavioral tests: Staggered Spondaic Words (SSW) for European Portuguese, Filtered Speech, Speech in Noise, Detection Interval in Noise, Duration, and Frequency Pattern. The normative data for children aged 5 to 12 are also reported. A sample was obtained of 217 subjects without ear pathology and with typical development. Each age group was composed of at least 30 children. All children were evaluated using pure tone audiometry, speech audiometry, impedance, and otoacoustic emissions. Normative scores are reported for each of the six auditory processing tests. The assessment is applicable to young children (aged 5 and 6). The statistical analyses showed significant effects in scores of Age for all tests and of Ear for several tests. The main result from the work presented, the Auditory Processing Assessment Battery—European Portuguese (BAPA-PE), is available for clinical use with normative data. This battery is a new tool for behaviorism assessment of European Portuguese speakers with suspected central auditory pathology and for monitoring the results of auditory training.

## 1. Introduction

Health professionals responsible for conducting hearing assessment have been experiencing an increasing number of requests related to patients with normal hearing sensitivity thresholds and in which the peripheral assessment is not enough to clarify the difficulties reported by individuals or family members. These situations require a more complete audiological assessment, including assessment of Central Auditory Processing Disorders (CAPD) [1]. There is also an increase in the of otorhinolaryngology (ORL) services and audiology departments regarding the assessment of children, especially those with learning difficulties due to suspected hearing disorders. These children often have behavior similar to that of those with alterations in peripheral auditory sensitivity, with a clear negative effect on academic performance but their basic audiological evaluation does not reveal any changes.

Auditory processing disorder is defined as a specific deficit in the processing of auditory information along the central auditory nervous system, including bottom-up and top-down neural connectivity [2,3] Since the middle of the 20th century (1950s), countless works have contributed to the development of tests and batteries for the evaluation of central auditory processing. Katz created the dichotic Staggered Spondaic Words (SSW) test in the early 1960s, which today is still a widely used test with a great capacity to contribute to the diagnosis of auditory processing disorders [4,5]. Several batteries were developed for other languages. A representative, small, selection is summarized in Table 1. Some were published after the development of the work presented in this paper.

The Central Auditory Processing assessment tests are categorized as low-redundancy monoaural tests, dichotic tests, temporal processing tests, and binaural interaction tests [1,15]. To allow evaluating individuals while controlling the influence of linguistic abilities and to evaluate the different mechanisms and processes of auditory processing, as well as different levels and regions of the central auditory pathway [2,16], a battery of evaluation tests should be composed of tests with verbal and non-verbal stimuli. These batteries of tests may include, but are not limited to, tests that evaluate the following processes: sound localization and lateralization, auditory discrimination, auditory temporal processing, auditory pattern processing, dichotic hearing, auditory performance with stimuli in competition and with degradation [3,15,17].

Although several international tests were created and validated for various languages, the assessment of central auditory processing in listeners who speak European Portuguese continues to require further investigations. Extensive studies are necessary to develop, improve and optimize a battery of tests applicable to the Portuguese population and native Portuguese speakers worldwide. The dispersion of Portuguese-speaking individuals in several countries around the world that cannot be assessed with batteries created for natives of the official languages of the host countries is another important motivation for the development of this battery. In general terms, there was a need to provide listeners who are native Portuguese speakers, and especially to younger age groups, the possibility of evaluating the Central Auditory Processing (CAP). Therefore, it is not enough to develop a set of auditory assessment tests: it is also necessary to obtain normative data for Portuguese speakers, including children. This battery, like those applied in other countries, should allow the collection of adequate information, aiming at the definition of a correct differential diagnosis as well as relevant guidance for individualized treatment [18].

The importance of these instruments lies, mainly, in the early detection of changes in the development of the central auditory system, allowing a qualitative and quantitative assessment of the auditory skills and enabling a timely intervention on the skills that perform below the expected level for each age group. Some international authors, in the development of their tests, already present data for the age groups of 5, 6, and 7 years, recognizing the influence of changes in auditory processing in school difficulties and alerting to the need to carry out the assessment at earlier ages [19]. Shapiro, in 2016, points to the need to reduce the age of assessment, recommending early assessment in order to anticipate the intervention as much as possible, to try to contribute to the reduction of school difficulties [20]. Assessment before entry into basic education, allowing early diagnosis and intervention, is defended by several researchers, namely the evaluation of abilities of sound localization and verbal and non-verbal sequential memory, as they are important processes whose development occurs in the first 6 to 7 years of life and which present themselves as of great importance in the language acquisition and development [21].

To summarize the key points presented before, the main motivations for the development of the battery and the computer application were: (1) the need in more and more situations to go beyond peripheral assessment (2) create conditions to assess (central) auditory processing as early as possible; (3) provide Portuguese native speakers with central auditory assessment at state-of-the-art level.

### Paper Structure

After the abovfe presentation of the motivation and need for the development of an Auditory Processing Assessment Battery for children native European Portuguese speakers, the following sections are structured as follows: Section 2 presents some related work; Section 3 presents information regarding the battery and its development; normative results obtained with a sample of more than 200 children and their analyses are the subject of Section 4; after discussion of the results, in Section 5, this paper concludes with main conclusions and the identification of relevant future work.

NOTE: Original Section 2 completely deleted. Information transformed into Table 1.

## 2. Methods

This section presents information regarding the developed battery, starting with test selection.

### 2.1. Selection of Tests for the Battery

Ince it was not possible to carry out the work with a large set of tests, work started by a selection process, selecting the tests most frequently used in daily practice [15,22] and tests and test categories most frequently used in recently developed European and International Auditory Processing (APD) batteries, according to the main test categories identified by the American Speech-Language-Hearing Association (ASHA) and the American Academy of Audiology (AAA) and with reported abilities to detect alterations in groups of children with problems such as learning difficulties.

The need to develop an evaluation battery that is as balanced as possible led to the choice of tests that would evaluate as much as possible all the areas of auditory processing. After consent was received for the battery should to tests with verbal and non-verbal stimuli [23], an equal number of tests of each of these types was selected.

The test selection process resulted in a battery consisting of the 6 following tests: 3 verbal tests (1 dichotic: SSW for European Portuguese; 2 monoaural low redundancy: Filtered Speech, Speech in noise); 3 non-verbal tests (temporal processing: Detection of Interval in Noise, Frequency Pattern, Duration Pattern).

### 2.2. Development of the BAPA-PE Battery

This section describes the 6 tests selected for the battery named BAPA-PE (from the Portuguese version of Auditory Processing Assessment Battery—European Portuguese), providing mainly information regarding their development. They resulted from a continuous line of work and are direct results of [5,24,25].

#### 2.2.1. SSW for European Portuguese (SSW-EP)

The SSW test for European Portuguese (SSW-EP) integrating the battery was developed by the authors in 2007 following the principles of the original test. Due to the syllabic structure of the Portuguese language, disyllabic words were used. The development included a careful process of selection of the words that involved linguists, elementary school teachers, and young children (5 and 6 years old) [25].

The final version of this test complies with all the characteristics of the original one, as checked by the author. (A screen example for this test is available as supplemental online material; see section at end of the paper).

The creation of the stimuli consisted of: (1) selection of a voice to record the word sequences; (2) recording in a studio; (3) annotation of the words “begin” and “end”; (4) creation in Matlab [26] of vectors for the two stereo channels making the necessary temporal adjustments to have the first word in one channel starting synchronously with the second word in the other; (5) saving the result as a stereo wave file.

This test creates different conditions for comprehension tasks on the part of the patient: (1) words heard without another sound competing for attention in the other ear, a condition called “Non-Competitive” (NC); (2) words heard simultaneously with others, the “Competitive” situation (C). As these conditions are applied to each ear, we get RNC (right ear non-competitive condition), RC (right ear competitive), LC (left ear competitive), and LNC (left ear non-competitive).

The test starts with the right ear and alternates the ear for the following stimuli, guaranteeing that half of them start the presentation in each ear.

The results kept for this test are percentage of correct answers and errors in each of the 4 conditions tested (RNC, RC, LC, LNC).

#### 2.2.2. Filtered Speech (FS)

The Filtered Speech test was developed with a list of 20 numbers (e.g., 8, 18, 31, 1000), recorded in Portuguese by a female speaker. The processing, made in Matlab, consisted in the application of a low-pass filter with a cutoff frequency of 1000 Hz [27].

During application of the test, each stimulus is presented twice randomly. The subject being evaluated is asked to repeat the numbers, with the number of correct repetitions being counted.

Before integrating the battery, this test had already been used in the evaluation of individuals with cochlear implants (adults and children); however, normative data had not yet been obtained for the test.

The results for this test are the percentage of correctly repeated numbers for each ear.

#### 2.2.3. Speech in Noise (SIN)

The Speech in Noise test was developed with 30 Portuguese disyllabic words, recorded with a female voice. To ensure the use of linguistic stimuli adequate for the evaluation of pediatric population, only commonly used, well-known words with CVCV structure were selected.

To create the stimuli, babble noise was added to the recording controlling the Signal to Noise Ratio (SNR). Two versions of the stimuli were created: one with SNR +10 dB and the other with SNR +15 dB. The resulting stimuli were presented randomly [24,27].

For integration in the battery, the initial version of the test, developed for use with cochlear implants (adults and children), was improved regarding the method used, we measured the word’s acoustic envelope and placed the noise so that the SNR would be the same throughout the word.

During test administration, the subject being evaluated was requested to repeat the words. The results for this test are the percentage of correctly pronounced words for each of the SNR conditions plus the global percentage when considering both conditions together.

#### 2.2.4. Detection of Interval in Noise (DIN)

In this test, 6 s of white noise with small silence intervals at random positions are presented to the subject. The subject answers by raising a hand when he/she detects a silence interval.

The first version of the test of Detection of Interval in Noise was developed in 2010 [24], having been initially applied to children and adults with cochlear implants [27]. These initial studies evidenced the need to create a new version of the test with the inclusion of new intervals of silence with shorter duration. As a result, silence intervals lasting 5, 7, 8, 9, 12, 16 ms were added, integrating the final test the following intervals: 2, 4, 5, 6.7, 8, 9, 10, 12, 14, 16, 18, 22, 26, 30 and 40 ms. Each stimulus was presented 6 times in each ear throughout the evaluation.

The stimuli were created in Matlab [26] and exported to .wav files.

In the development of this test, a major effort was made to simplify the task of the test administrator, resulting in the creation of a novel form of presenting all the test information. The application presents information about the number of intervals, their temporal position and duration. (A screen example for this test is available as supplemental online material, see section at end of the paper). In addition to the numbers, the position of the intervals is indicated by 3 sliders that are positioned at the appropriate instant. A fourth slider, the first, moves in sync with the stimulus hearing, facilitating the detection of correct or incorrect answers.

The results for this test are the percentage of correctly detected intervals and hearing threshold (smaller duration of the silence intervals with percentage of detection not inferior to 50%) for each ear.

#### 2.2.5. Frequency Pattern (FP)

The Frequency Pattern test was developed in 2010 [24], being a variation of the initial test of [28]. The test developed includes sequences of three and four 200 ms segments of two different frequencies (800 and 1300 Hz), with intervals between segments of 250 ms. The total number of sequences is 34 (10 of three segments and 24 of four segments), which were created in Matlab [26] and exported to wave files. The individual under evaluation was asked to name the sound sequence heard (e.g., high–low–low–high) [24,27].

This test was initially used in the evaluation of individuals with cochlear implants (adults and children), with no normative data for the test in the Portuguese population at the time of inclusion in the battery.

From this test are obtained, as results, for each ear, the percentage of correctly reproduced sequences by the subject being assessed, for stimuli of 3 segments, stimuli of 4 segments, and all 34 stimuli.

#### 2.2.6. Duration Pattern (DP)

The Duration Pattern test was also developed in 2010 [24] having as basis the work of Baran and coworkers [29]. The test developed integrates 11 sequences of 3 segments and 23 sequences of 4 segments, with segment durations of 300 ms (short segments) and 600 ms (long segments) [24,27]. All segments were pure tones of 1000 Hz. An interval (silence) of 250 ms separates each of the segments of the stimuli.

This test had already been used in the evaluation of individuals with cochlear implants (adults and children); however, normative data had not yet been obtained for the test in the Portuguese population.

As for the Frequency Pattern, for each ear, the percentage of correctly reproduced sequences were obtained as results by the subject being assessed, for stimuli of 3 segments, stimuli of 4 segments, and all 34 stimuli.

### 2.3. Application to Normal Hearing Group

**Figure 1:**
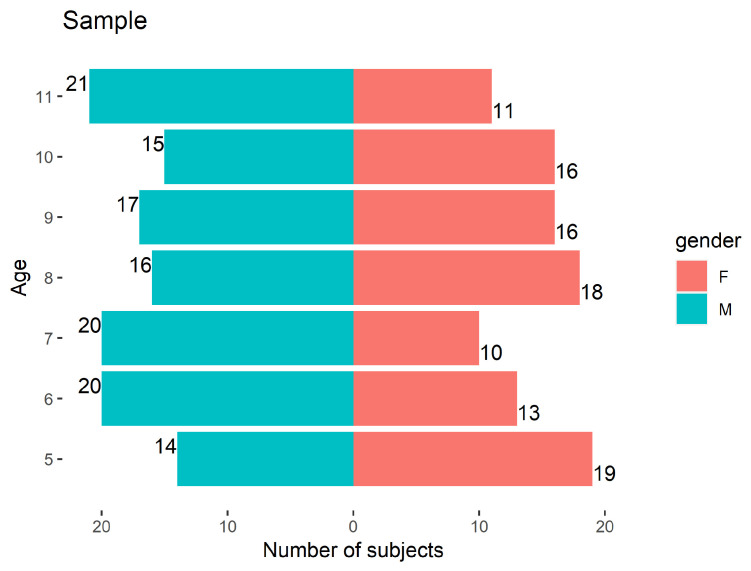
Sample distribution by age and gender.

The normative values result from the application of the battery to a group of children without ear pathology and with typical development, within the scope of the first author’s PhD [5]. The sample, planned to contemplate at least 30 subjects per age groups, was collected in 13 locations across Portugal (covering the four regions: North, Center, South and Islands) and is composed of 217 children without pathology, 116 male (53.5%), distributed among the 7 age groups, between 5 and 12 years old (exclusive), as shown in Figure 1. The distribution of the children in the sample according to laterality was 204 (94.0%) right-handed, 12 (5.5%) left-handed, 1 (0.5%) ambidextrous.

As inclusion criteria were considered all normal hearing individuals without a history of ORL, neurological and psychological pathology, who did not present changes in the battery of audiological tests performed. The criteria adopted for normal hearing are presented in Table 2.

All data were collected with the authorization of the responsible entities, the individuals studied and/or legal representatives of the children studied (free and informed consent), ensuring total confidentiality of the data obtained.

For the presentation of the tests, a computer application was developed allowing the application of the tests and the automatic creation of the record sheet with the responses of the evaluated patient. Evolution of this application resulted in a commercial product in use by several clinics and hospitals [30].

The tests were presented at 50 dB HL in which the application of monoaural tests was 50% first in the right ear and 50% first in the left ear, in order to reduce the learning effect.

#### Analyses

The results obtained by the children were subjected to descriptive statistical analyses (mainly average values, standard deviation and their variation with several factors) and statistical tests, performed in R and SPSS. The non-normal distribution of the results (originally counts of correct answers except for DIN test thresholds) was considered, resulting in the adoption of Generalized Linear Models (GLM). GLM models were performed using the function glm() in R [31] with a Quasi-Poisson distribution for cases with variance larger than the mean (sign of overdispersed count data) and Poisson distribution for the other cases (Poisson used only for the threshold of the SIN test). Information regarding the GLM models used is presented in Table 3. The significance level (α) adopted was 0.01 and the residuals used to assess the models.

## 3. Results

In this section, the results will be presented for the six tests that made up the battery of tests in the representative sample of children from 5 to 12 years old without pathology. In unilateral tests, the results are presented separately for each ear. The results are first presented globally. In the following sections, we will take an individualized and more detailed approach to the results in each of the tests performed. Additionally, detailed normative results for the six individual tests of the battery are available online (information in Supplementary Materials section at the end of the paper).

### 3.1. General Characterization

The average and standard deviation (in parenthesis) for the percentage of correct answers for the six tests were SSW 93.7 (4.7), FS 65.5 (17.6), SIN 58.8 (16.2), DIN 81.8 (6.6), FP 60.6 (31.6), DP 51.9 (29.3), showing that the performance in the tests is not homogeneous; there are some that have an average result of high performance, while others have lower average results. Furthermore, standard deviation is test dependent, with higher values for FP and DP. Only two tests achieved averages above 70%. Overall, the Duration test proved to be the most difficult, with an overall average of only 51.9% and SSW the test with the best results.

**Figure 2:**
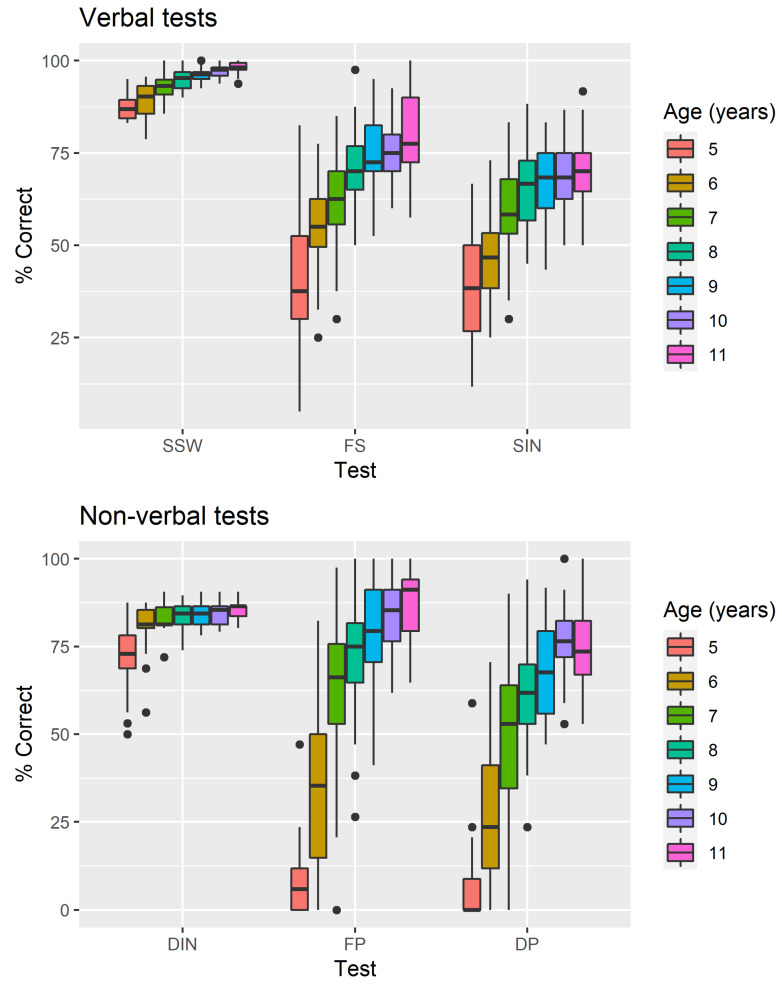
Boxplots for the percentage of correct answers for the 6 tests of the battery by age group. For monaural tests, only results for right ear are presented.

As the age group is the most relevant factor for this study, due to its effect on neuro-physiological maturation and, therefore, on the performance of the requested tasks, it is important to analyze to what extent it affects the overall results presented. Thus, we proceed to analyze the average percentage values of correct answers by age group, presented in Figure 2. For easier understanding, tests were split into two groups: verbal and non-verbal.

By analyzing the graphs in the figure, the effect of age on the performance of the requested tasks is clearly seen, with a lower performance manifested by the group of children aged between 5 and 6 years and an increasing performance in the different groups until the group of children aged over 11 years. The difference in performance is less evident in some tests (e.g., Detection of Interval in Noise). It is also notable that the group of younger children showed the greatest difficulty in the performance of the Duration Pattern and Frequency Pattern tests, with average percentages of correct answers approaching zero.

**Figure 3:**
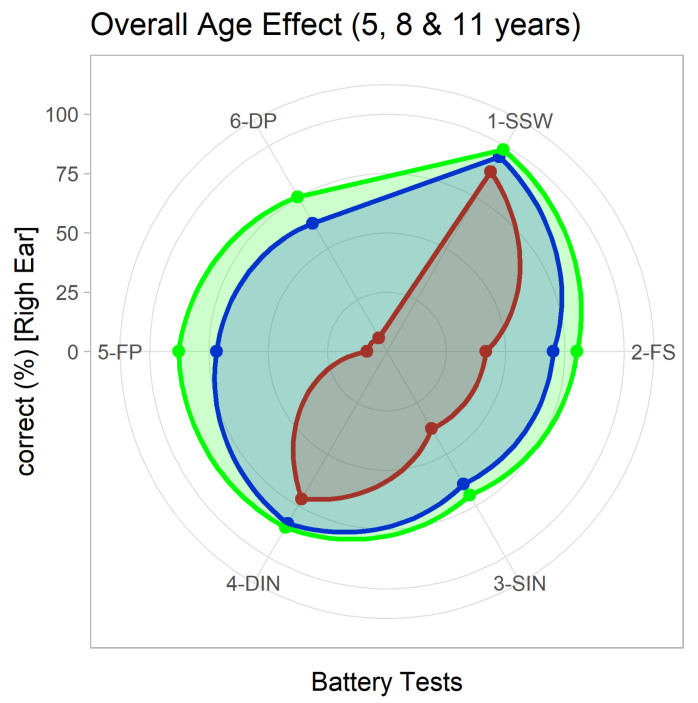
Simultaneous representation of the average percentage of correct answers for the 6 tests of the battery using a Radar plot. Values for 3 ages are presented: 5 years (in red), 8 years (blue) and 11 years (green).

The overall battery results can be better depicted in a multidimensional graphic, such as the radar plot. In Figure 3, to illustrate the effect of age in the complete battery, results are presented for three age groups (5, 8, 11).

The graphic shows that: the pattern of results for 5 years differs considerably from the other 2 higher ages, being much more asymmetrical due to the large differences in performance for individual tests; values for 9 years approach the ones for 11 years in general; as age increases the performance becomes more balanced (approaching a circle), but SSW, with consistent higher percentage of correct answers, causes a distortion; Frequency and Duration Pattern tests are the ones that have a higher age effect.

The results of GLM for the monaural tests confirmed the factor Age to be significant (p<0.001) and revealed as non-significant (p>0.01) Ear, Gender, Region, and Laterality effects. Similar results were obtained for the correct answers of the binaural test SSW, analyzed separately.

### 3.2. Results for Individual Tests

As the difference in the results of several tests is relevant, a more detailed analysis of the values obtained for each one was carried out. The first to be presented is the verbal tests, starting by the dichotic tests (SSW), then the low redundancy monoaurals (Filtered Speech and Speech in Noise). The nonverbal ones are presented after, following the order Detection of Interval in Noise, Frequency Pattern, and Duration Pattern.

**Figure 4:**
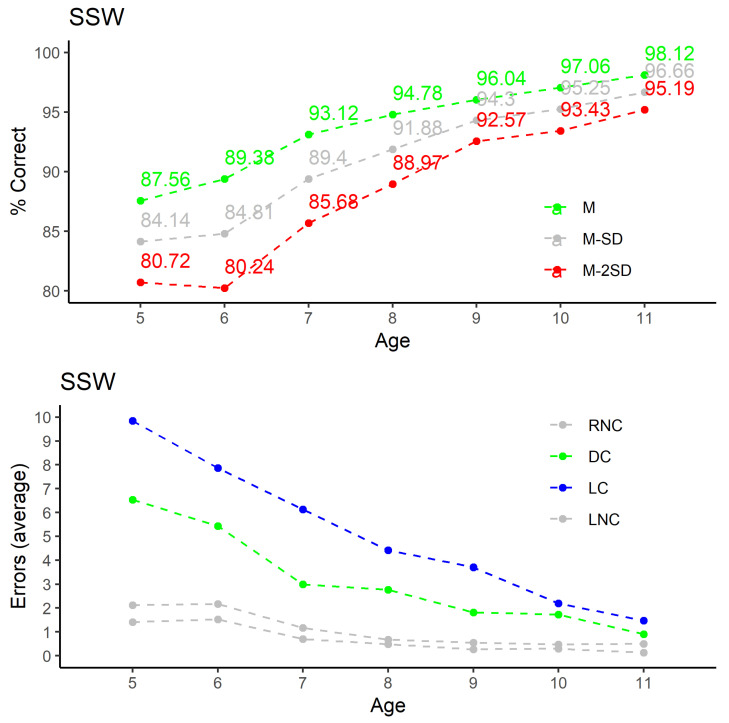
Results for the SSW test: (**top**) percentage of correct answers by age; (**bottom**) variation with age of average values for RNC, RC, LC, and LNC.

**Figure 5:**
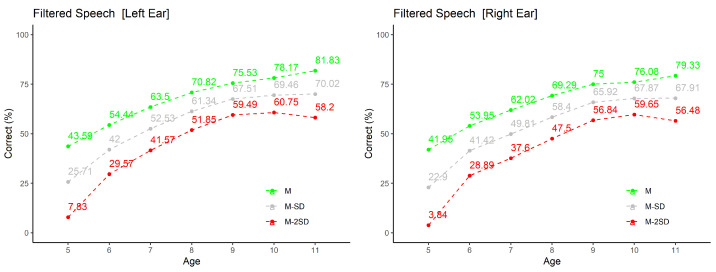
Results for Filtered Speech test: percentage of correct answers obtained in each ear by age group. In addition to the average value (M), M − SD and M − 2SD are shown.

**Figure 6:**
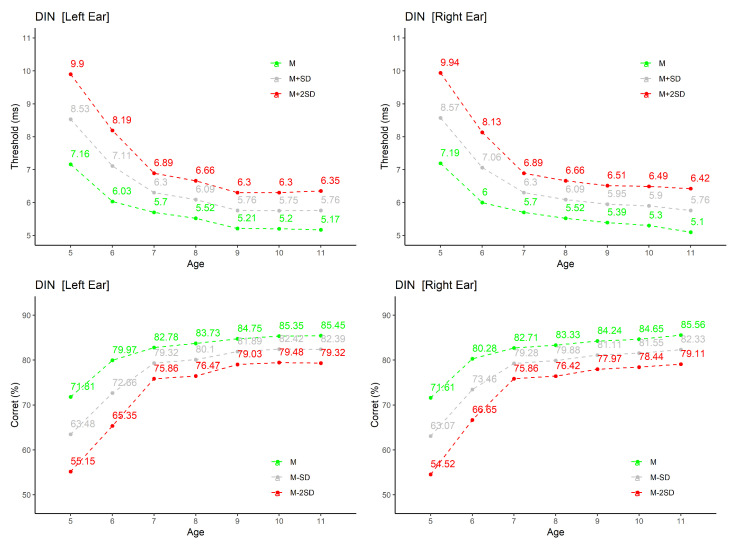
Results for Detection of Interval in Noise test: top row presents the hearing thresholds (in ms) by age group and ear; bottom row the percentage of correct answers also by age group and ear. In addition to the average value (M), M + 1SD and M + 2SD are shown for the threshold and M − 1SD and M − 2SD for correct answers.

A representative sample of the results is presented in graphical form in Figure 4, Figure 5 and Figure 6; the other are available in tabular format as supplemental material online. The selection includes two of verbal tests developed for EP.

In both cases—graphical representation and tables—are presented the average values (M), and averages plus/minus 1 and 2 standard deviations (M ± SD and M ± 2SD).

#### 3.2.1. SSW for European Portuguese

Although we previously presented the results of this test by the percentage of correct answers, we will now adopt the analysis of the percentage of errors, following the presentation of the results of the author of the SSW test in its original version. Thus, it is simpler to compare the results obtained in our sample with the normative data obtained for the original version of the test. As there is variation in the performance result according to the age group, we start by the effect of age in the SSW results, in Figure 4.

From the results, it is clear that: (1) much more errors occur for competitive situations, as expected, being higher for left ear; (2) errors in non-competitive situations are very low for ages above 7 years; (3) the errors in competitive situations show a continued decrease.

The GLM applied to the number of correct answers for the four test conditions (RNC, RC, LC, and LNC) showed significant effect of Age, Ear and existence of Competition, all with p<0.001.

#### 3.2.2. Filtered Speech

The graphics in Figure 5 show the values of the total percentage of correct answers as function of age group, separately for each ear. By analyzing them, it can be noticed that there is an increase in the average values of correct answers with increasing age, a reduction in dispersion of results with age, except for the final age group (from 11 to 12 years).

GLM results for this test confirm as significant the effect of Age (p<0.001). Ear effect is non-significant (p=0.247).

#### 3.2.3. Speech in Noise

For this test, a clear decrease is seen in results dispersion and an increase in the percentage of correct answers with increasing age.

Furthermore, very importantly, it is possible to observe that in the 5 years age group, the performance is low, but with M − 2SD above zero, not preventing its application in this age group.

Only the significance of the effect of Age was confirmed by GLM, with p<0.001. Ear effect is non-significant (p=0.197).

#### 3.2.4. Detection of Interval in Noise

The results for this test are summarized in Figure 6. The graphics in the figure present, separately, for each ear and by age group, the average thresholds in ms (upper part) and percentage of correct answers (lower part).

By analyzing the two graphs related to the thresholds, it can be seen that children in the age group from 5 to 6 years old have an average performance well below that of children in the remaining 6 groups, with average values for the threshold of approximately 7.4 ms for both ears. As age increases, the thresholds approach average values of approx. 5.1 ms, for both ears.

In the graphs with the percentages of correct answers, an increase in the values can be seen (from an average of around 72% at 5 years old to over 85% from 11 years old), as well as a reduction in the dispersion of results with increasing age. The increase in correct answers and reduction of hearing threshold with age is much more accentuated from 5 to 7 years. For ages 9 and above, the values remain quite stable. In the group of children with younger ages, the percentage of correct answers does not attain values below 50% for all subjects, justifying applicability of this test to this age group.

For thresholds and percentage of correct answers, the results obtained are very similar for both ears.

GLM results were the following: the effect of Ear is non-significant for both the percentage of correct answers (p=0.679) and hearing threshold (p=0.873); the effect of Age is significant for both factors (p<0.001).

#### 3.2.5. Frequency Pattern

The effect of age on test performance is once again clear. This test has very low values in the percentage of correct answers for children between 5 and 7 years old—with M − 2SD and even M − SD assuming the value 0—which show the difficulty of these tests for these children. The three-segment sequences show better results, showing positive values for M − 2SD for 6 years. To children 5 and 6 years old, only sequences of three stimuli are applicable. The increase in performance, similar for both ears and for the sequences of 3 and 4 segments, is accentuated up to the age of 9, being smoother thereafter. Average values for children aged 11 years and over are close to 90% correct, with percentages for three-segment stimuli above the 93%.

For this test, GLM confirms as non-significant the effect of Ear (p=0.841) and significant the effect of Number of Segments and Age (p<0.001 for both).

#### 3.2.6. Duration Pattern

The results for this test are similar to those obtained for the Frequency Pattern test: the effect of age on test performance is once again clear: very low values are presented in the percentage of correct answers for children between 5 and 7 years old; the sequences of three segments show better results, but only reach positive values for M − 2SD for the 7 years (in contrast to the 6 years of the Frequency Pattern test).

Like the Frequency Pattern test, the increase in performance, similar for both ears and for the sequences of three and four segments, is accentuated up to the age of 9, being smoother thereafter. The average values for children aged 11 years or over exceed 70% for four-segment stimuli and 80% for three-segment stimuli.

Similarly to the Frequency Pattern test, GLM confirms as non-significant the effect of Ear (p=0.994) and significant the effect of Number of Segments and Age, both with p<0.001.

## 4. Discussion

The battery of CAPD evaluation tests created proved to be applicable to the group of children aged between 5 and 12 years, and it was possible to obtain normative data for the Portuguese population, with special relevance for the tests in which there are no normative data in the international literature.

The results obtained for each age group corroborate the effect of the individual’s maturation on the performance of the requested tasks, which is in accordance with the international literature and justifies the need to obtain normative data for each age, especially in younger children.

Despite the variety of tests integrating batteries for the evaluation of central auditory processing, as shown in Table 1, making detailed comparisons difficult, the BAPA-PE battery integrates a number of tests, six, equal to the average number of tests of the batteries in the table and integrates tests similar to the ones of other batteries (e.g., Gaps In Noise, Duration, and Frequency Pattern, of The Norwegian Battery [13]).

The sample used, despite being smaller than the ones used for SCAN-C [7] and STAP [12], is close to the average for the representative batteries presented in Table 1 and higher than the sample used with the more recent battery in the table (Danish battery [14]), with results published in 2017. The 5 years adopted as the inferior limit for the age range is not a common choice. All but the sample used for SCAN-C [7] have ranges starting at 6 years or higher.

The results for each test are discussed in the following subsections.

### 4.1. SSW for European Portuguese

After conducting this research and processing the data collected with the application of the EP SSW test, we can conclude that the data obtained in our work are within the confidence intervals of the works published by Katz [4]. The values obtained in the present study have a mean and standard deviations smaller than those published by Katz, which make it more sensitive in detecting pathology. These results may result from the fact that the limits of normality obtained in the original test were considered as a factor for inclusion in the sample.

### 4.2. Filtered Speech

When comparing the data relating to children with ages between 7 and 12 years of the present study with those obtained by Bellis [32], in Table 4, the performance in each age group age is similar in the two studies.

Data were also obtained for children aged 5 and 6 years, extending the applicability of the test. The results obtained were: 42% and 44% of correct answers for the right and left ear, respectively, for children of 5 years, and 54% for both ears for children 6 years old.

The data presented in this work, in addition to showing the performance in each ear separately, includes data for younger age groups, which in itself will foster the implementation of intervention plans earlier.

### 4.3. Speech in Noise

The tests referred to in the literature use monosyllabic stimuli. The test of this investigation uses disyllabic stimuli, a different mask sound (Babble) and SNR of +10 and +15 dB. These facts may make the test of the present work more applicable. However, after processing the collected data, it was found that this task represented some difficulty for the children evaluated, even those of the older age groups. Thus, by comparing the data from the present study with the data obtained in the work carried out by Mangabeira-Albernaz [33], it was verified that the 70% correctness barrier, referred to there as a normal limit, which is only reached in this study by the group of 11-year-old children. This circumstance is due to the fact that the test used consists of half of the stimuli with an SNR of +10 and the other half of stimuli of SNR +15, which makes the whole of the test more difficult.

### 4.4. Detection of Interval in Noise

Several authors presented normative values for this test. Taking as a reference the data presented by the original author of the test in 2009 [34], the results obtained in this study are, on average, superior to those found in the original work, although they have less variance.

The results obtained for 5 and 6 years, with average values similar to the value obtained with 2 SD in 7-year-old children, indicates that this test can provide useful information, justifying its application in these age groups and allowing earlier identification of children at risk.

Regarding the comparison of the percentage values of correct answers, it should be noted that the data presented in the present work will necessarily have to be different, since the test used consists of more intermediate intervals, which will cause changes in the mean values and standard deviations.

### 4.5. Frequency Pattern

The data in the literature refer to the original test, which is formed only by sequences of three stimuli. The results of the present work originate from a test that consists of sequences of three and four stimuli. This fact makes performance even more difficult, particularly in younger children. As we evaluated children aged 5, 6, and 7 years old, which, according to Musiek in 2006 [35], are not suitable for the application of this assessment, we could verify that, comparing the data that refer to the sequences of three stimuli, the test can be applied without much difficulty for children aged 7 years.

The results of total performance observed in our work show that it is possible to apply it to children aged 6 and 7 years. At the limit, 6-year-olds can be assessed considering only the stimuli of three segments.

### 4.6. Duration Pattern

As in the previous test, the data for the original test result from the presentation of sequences of three stimuli. In the present work, the results come from a test that consists of sequences of three and four stimuli. The observation that was made for the previous test applies to this one. It can be applied without great difficulty to children 7 and 8 years old. At the limit, 6-year-olds can be assessed considering only the sequences of 3 stimuli.

## 5. Conclusions

Answering the need of adequate state-of-the-art tests for European Portuguese native speakers, this paper presents a battery for Central Auditory Processing assessment, named BAPA-PE, providing information from tests selection and development to normative data. This battery is a contribution to the state of the art in hearing assessment of European Portuguese speakers, providing a tool that allows conducting behavioral assessments of individuals with pathology of the central auditory system.

The normative data for children aged 5 to 12 are also reported. These data are of primary importance for the clinical evaluation of individuals who have normal hearing thresholds but have difficulties in hearing performance in unfavorable listening situations, who, in this way, can see their results compared with individuals without pathology. The obtained normative values include for the first time normative data for children of 5 and 6 years of age in some of the tests, filling a gap in the literature and demonstrating the applicability of the battery to ages as low as 5 years, fostering earlier referencing of risk situations.

Despite being developed for European Portuguese native speakers and normative values obtained with children living in Portugal, the battery has potential to be used in other situations, such as: (1) the many countries around the world with communities of native speakers of European Portuguese (e.g., Luxembourg, France, USA, Brazil); (2) other Portuguese speaking countries and communities in Africa (e.g., Angola) and Asia (e.g., Macau), typically with dialects closer to those spoken in Portugal than in Brazil (with caution regarding the normative values). Furthermore, the three language-independent non-verbal tests and their normative values can be useful to form the basis for new batteries for other languages—complemented by verbal tests—or to make possible partial auditory assessment of children in languages not covered by existing batteries.

The combination of the battery with the normative data created the conditions for the development of a computational application integrating all battery tests and allowing its simple use by audiologists, existing as a commercial product since 2018 [30]. Besides tests administration, the application integrates contributions related to the best way to present the information to the evaluator—with the test of detection of interval in noise being a good example—and in the form of providing results (detailed report, but at the same time, simple analysis).

### Future Work

After the development of the battery and its application to children without pathologies, this work was continued (and is being continued) in several directions: (1) exploration of the correlation of tests results to investigate a smaller battery suitable for early screening of auditory processing [5]; (2) application to subjects aged 12 and above; (3) application of the tests to children and adults with comorbilities (e.g., learning difficulties [5]); (4) application to children and adults with cochlear implants; (5) development and commercialization of an application [30].

Currently, this battery is being used in clinical practice to evaluate children with suspected APD and, if the diagnosis is confirmed, to evaluate the development of the skills after auditory training. Furthermore, this battery is being used to evaluate a group of children with learning difficulties, who do not have other associated pathologies, such as attention deficit, hyperactivity, etc., and we will soon publish the results of the study.

## Figures and Tables

**Table 1:** A representative small selection of batteries for the evaluation of central auditory processing.

Battery	Ref	Information
SCAN-A: Test of Auditory Processing Disorders in Adolescents and Adults	[6]	Can be seen as an early example of a battery. Six tests: two filtered words (FW), two auditory figure-ground (AFG), a competing words (CW), and a competing sentences (CS).
SCAN-C: Test for Auditory Processing Disorders in Children-Revised	[7]	The improvements to the initial test included new test instructions to make them easier for young children, stimuli presented on a compact disc, revision of the Competing Words subtest, and the addition of a Competing Sentences subtest. Normative data were obtained on **650 children** age 5 years to 11 years, 11 months old and published in 2000.
MAPA: Multiple Auditory Processing Assessment	[8]	Developed based on Musiek and Chermak [9] and ASHA recommendations [10] and published in 2000. Includes four commonly used tests, selected and recorded for use in assessing school children. The tests were administered to a sample of **81 third grade children** along with the SCAN. Four separate factors emerged from the four MAPA tests: monaural separation/closure, auditory pattern/temporal ordering, binaural integration, and binaural separation.
Dutch battery	[11]	Originally developed to diagnose auditory processing disorders (APDs) in adults. Consists of eight tests: words-in noise, filtered speech, binaural fusion, dichotic digits, frequency and duration patterns, backward masking, categorical perception and digit span. Descriptive statistics were computed in 2002 on data obtained from **75 children** from primary school (age 9–12 years) and 30 adolescents from secondary school (age 14–16 years) with normal hearing and intelligence. Age effects were present in most tests.
STAP: Screening Test for Auditory Processing	[12]	Contains four subsections: speech-in-noise, dichotic consonant vowel, gap detection and auditory memory. It was administered to **500 school-going children** in the age range of 8–13 years (141 children at-risk on the Screening Checklist for Auditory Processing).
Norwegian Battery	[13]	Consists of Filtered Words, Competing Words, Dichotic Digits, Gaps In Noise, Duration and Frequency Pattern, Binaural Masking Level Difference, and HIST Speech in Noise test. A total of **268 normal hearing children** aged 7–12 years participated in the study. Results, published in 2018, revealed no differences between genders. The children showed improving performance by age on all tests, except for the Gaps In Noise and Binaural Masking Level Difference.
Danish Auditory Processing Disorders (APD) battery	[14]	Published in 2017. Consists of four behavioral tests: the filtered words (FW) test, the dichotic digits (DD) test, the gap detection (GD) test, and the binaural masking level difference (BMLD) test. Evaluated on **158 children** (75 boys and 83 girls, aged 6–16 years) with no known history of auditory problems to obtain normative values.

**Table 2:** The criteria adopted for normal hearing.

Test	Normality Criterion
Tonal audiogram	20 dB HL at 125, 250, 500, 1000, 2000, 4000, 8000 Hz .
Bilateral tympanogram	Type A curve, ipsi and contralateral acoustic reflexes present within the normal range. (Acoustic admittance peak 0.35 to 1.25, mm H_2_O—between −50 to + 50 daPa.)
Otoacoustic emissions	Present within the normal range (DPOAE—F1 65 dB SPL, f2 50 dB SPL, SNR at 4/5, f2 frequencies 3dB higher than 2 standard deviations above the mean noise).

**Table 3:** Information regarding the Generalized Linear Models (GLM) models used for the analyses of the auditory tests.

Analysis	Sec.	GLM Model
All Monaural	Section 3.1	correct Test + Gender + Age + Ear + Region + Laterality + (1| Subject)
SSW (global)	Section 3.1	correct Gender + Age + Region + Laterality + (1| Subject)
SSW	Section 3.2.1	correct Age + Ear + Competition + (1| Subject)
FS	Section 3.2.2	correct Age + Ear + (1| Subject)
SIN	Section 3.2.3	correct Age + Ear + (1| Subject)
DIN	Section 3.2.4	threshold Age + Ear + (1| Subject)
		correct Age + Ear + (1| Subject)
FP	Section 3.2.5	correct Age + Ear + numSegments + (1|Subject)
DP	Section 3.2.6	correct Age + Ear + numSegments + (1|Subject)

**Table 4:** Comparison of results obtained for the filtered speech test with normative data of Bellis (2003) for the Auditec test.

Age	Bellis (2003)	Left	Right
7	62	64	62
8	70	71	69
9	68	76	75
10	72	78	76
11	75	81	79

## Data Availability

Data is contained within the article or supplementary material.

## Supplementary Materials

The following are available online: https://www.mdpi.com/article/10.3390/audiolres11030044/s1, Figure S1: Screen example for SSW test for European Portuguese, showing information presented during test administration (https://bit.ly/3yP8rnm, accessed on 14 September 2021), Figure S2: Screen example for the test of Detection of Interval in Noise, showing information presented during test administration (https://bit.ly/2Tw3BeR, accessed on 14 September 2021), Tables: Detailed normative results for the 6 individual tests of the battery (https://bit.ly/2Vaj4BV, accessed on 14 September 2021).
