# Supplementary material for: Auditory Processing Disorder Test Battery in European Portuguese—Development and Normative Data for Pediatric Population
Source: Audiol Res. 2021 Sep 17;11(3):474–90. doi: 10.3390/audiolres11030044 (PMC8482123; doi:10.3390/audiolres11030044)

Auditory Processing Disorder Test Battery in European Portuguese: Development and Normative Data for Pediatric Population

Annex I - Screen example for SSW test for European Portuguese, showing information presented during test administration.

The Figure shows how the information regarding the correct words is presented to the test administrator. In the example shown the stimuli consists in “Leite Branco” (White Milk) and “Sopa Quente” (Hot Soup), one presented to the left ear and the other to the right ear.

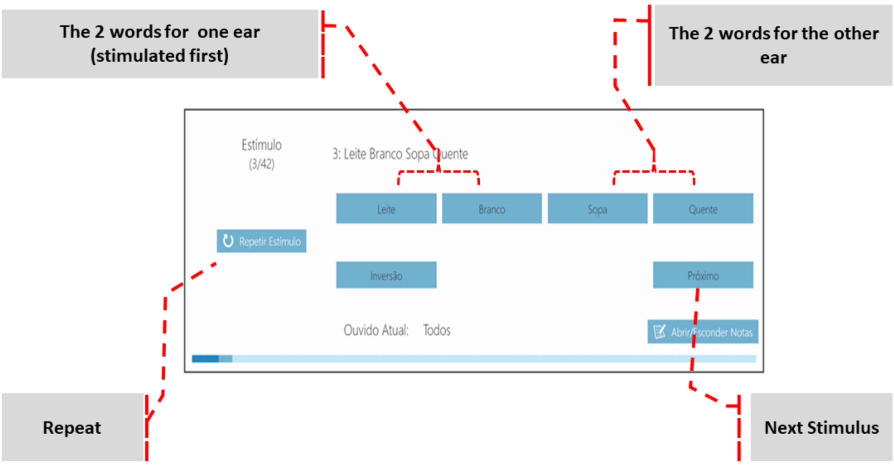

Supplement: Supplementary file 1 [file audiolres-11-00044-s001.zip › Supplemental1.pdf]
