# Supplementary material for: Auditory Processing Disorder Test Battery in European Portuguese—Development and Normative Data for Pediatric Population
Source: Audiol Res. 2021 Sep 17;11(3):474–90. doi: 10.3390/audiolres11030044 (PMC8482123; doi:10.3390/audiolres11030044)

Auditory Processing Disorder Test Battery in European Portuguese: Development and Normative Data for Pediatric Population

Annex II - Screen example for the test of Detection of Interval in Noise, showing information presented during test administration.

In the development of this test, a major effort was made to simplify the task of the test administrator, resulting in the creation of a novel form of presenting all the test information, shown in the Figure.

The application presents information about the number of intervals, their temporal position and duration. In the Figure is presented an example with 3 intervals, the first of 30 ms starting at 749 ms, the second, with a duration of 9 ms, starting at 4233 ms, and the third starting at 4758 ms and having a duration of 14 ms.

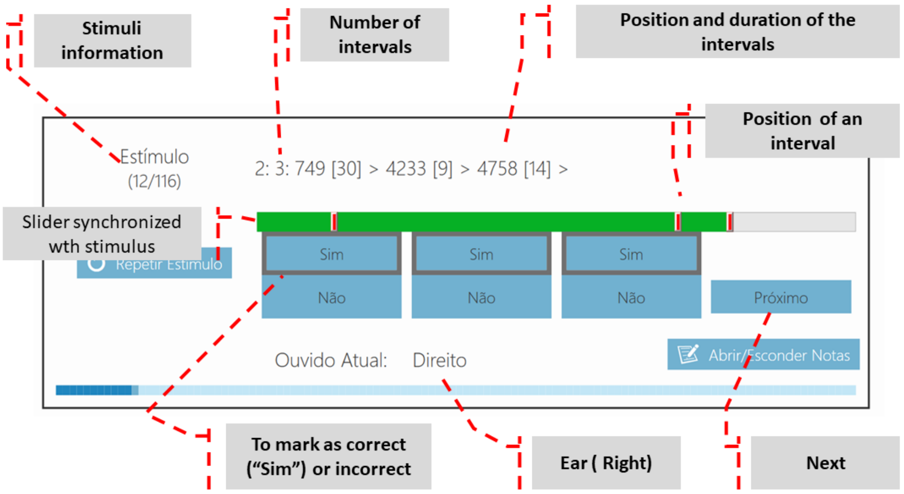

Supplement: Supplementary file 1 [file audiolres-11-00044-s001.zip › Supplemental2.pdf]
