# Supplementary material for: Auditory Processing Disorder Test Battery in European Portuguese—Development and Normative Data for Pediatric Population
Source: Audiol Res. 2021 Sep 17;11(3):474–90. doi: 10.3390/audiolres11030044 (PMC8482123; doi:10.3390/audiolres11030044)
Supplement: Supplementary file 1 [file audiolres-11-00044-s001.zip › Supplemental3.pdf]

Auditory Processing Disorder Test Battery in European Portuguese:
Development and Normative Data for Pediatric Population

Annex III - Detailed results for the 6 individual tests of the battery.

Table 1: SSW results for children native speakers of European Portuguese aged 5 to 12 years: Number of errors for each of the test conditions and total percentage of correct answers for each age group, in the group of children without pathology. In addition to the average value, Average+1SD and Average+2SD for errors and Average-1SD and Average-2SD for the percentage of correct answers are shown.

**Age**		**RNC**	**RC**	**LC**	**LNC**	**% Cor**	**N**
5	M	1.41	6.53	9.84	2.12	87.56	32
	M±SD	2.28	9.21	12.89	3.39	84.14	
	M±2SD	3.16	11.88	15.94	4.65	80.72	
6	M	1.53	5.43	7.87	2.17	89.38	31
	M±SD	2.73	8.07	11.52	3.68	84.81	
	M±2SD	3.92	10.70	15.18	5.19	80.24	
7	M	0.70	3.00	6.13	1.17	93.12	30
	M±SD	1.54	5.35	9.67	2.19	89.40	
	M±2SD	2.37	7.70	13.21	3.21	85.68	
8	M	0.48	2.77	4.42	0.68	94.78	31
	M±SD	1.21	4.68	6.81	1.51	91.88	
	M±2SD	1.93	6.59	9.20	2.34	88.97	
9	M	0.27	1.82	3.70	0.55	96.04	33
	M±SD	0.72	3.00	5.35	1.11	94.30	
	M±2SD	1.18	4.19	6.99	1.67	92.57	
10	M	0.30	1.73	2.20	0.47	97.06	30
	M±SD	0.77	2.75	3.93	1.04	95.25	
	M±2SD	1.23	3.76	5.66	1.61	93.43	
11	M	0.13	0.90	1.47	0.50	98.12	30
	M±SD	0.57	1.56	2.75	1.32	96.66	
	M±2SD	1.00	2.22	4.03	2.14	95.19	

Table 2: Results for Filtered Speech for children native speakers of European Portuguese aged 5 to 12 years: Percentage of correct answers for each age group for both ears are presented. In addition to the average value, Average-1SD and Average-2SD are shown.

Age		**Right Ear**	**Left Ear**	N
5	M	41.95	43.59	32
	M−SD	22.90	25.71	
	M−2SD	3.84	7.83	
6	M	53.95	54.44	31
	M−SD	41.42	42.00	
	M−2SD	28.89	29.57	
7	M	62.02	63.50	30
	M−SD	49.81	52.53	
	M−2SD	37.60	41.57	
8	M	69.29	70.82	31
	M−SD	58.40	61.34	
	M−2SD	47.50	51.85	
9	M	75.00	75.53	33
	M−SD	65.92	67.51	
	M−2SD	56.84	59.49	
10	M	76.08	78.17	30
	M−SD	67.87	69.46	
	M−2SD	59.65	60.75	
11	M	79.33	81.83	30
	M−SD	67.91	70.02	
	M−2SD	56.48	58.20	

Table 3: Speech in Noise results for children native speakers of European Portuguese aged 5 to 12 years: Percentage of correct answers for each age group for both ears and the 2 SNR conditions are presented. In addition to the average value, Average-1SD and Average-2SD are shown.

		**Right Ear**			**Left Ear**			
Age		SNR=+10	SNR=+15	Total	SNR=+10	SNR=+15	Total	N
5	M	30.83	44.38	37.60	31.77	47.19	39.43	32
	M−SD	16.12	26.65	21.70	17.58	28.24	23.40	
	M−2SD	1.41	8.92	5.80	3.39	9.28	7.38	
6	M	39.77	53.34	46.53	41.41	54.84	48.11	31
	M−SD	27.70	40.62	35.21	27.64	42.35	35.83	
	M−2SD	15.62	27.89	23.89	13.88	29.85	23.56	
7	M	53.99	64.88	59.27	54.69	66.11	60.67	30
	M−SD	40.18	51.04	46.62	40.68	52.05	47.36	
	M−2SD	26.38	37.21	33.98	26.67	37.98	34.06	
8	M	56.67	71.40	64.16	59.03	70.85	65.05	31
	M−SD	45.03	59.36	53.04	45.86	57.73	52.84	
	M−2SD	33.38	47.31	41.92	32.69	44.61	40.63	
9	M	57.07	73.64	65.35	57.78	73.33	65.56	33
	M−SD	45.38	60.91	54.17	46.12	60.67	54.22	
	M−2SD	33.69	48.17	43.00	34.46	48.00	42.89	
10	M	63.22	75.78	69.44	65.11	76.33	71.00	30
	M−SD	50.11	67.57	60.17	52.34	67.48	61.64	
	M−2SD	36.99	59.36	50.89	39.58	58.62	52.28	
11	M	63.88	74.89	69.71	67.01	75.78	71.39	30
	M−SD	53.55	63.41	59.56	55.97	65.50	61.53	
	M−2SD	43.23	51.94	49.41	44.93	55.22	51.68	

Table 4: Results for Detection of Interval in Noise for children native speakers of European Portuguese aged 5 to 12 years: Percentage of correct answers and hearing threshold for each age group are presented for both ears. In addition to the average value, Average+1SD and Average+2SD are shown for the threshold and Average-1SD and Average-2SD for percentage of correct answers.

		**Right Ear**		**Left Ear**		
Age		Threshold (ms)	Correct (%)	Threshold (ms)	Correct (%)	
5	M	7.19	71.61	7.16	71.81	32
	M±SD	8.57	63.07	8.53	63.48	
	M±2SD	9.94	54.52	9.90	55.15	
6	M	6.00	80.28	6.03	79.97	31
	M±SD	7.06	73.46	7.11	72.66	
	M±2SD	8.13	66.65	8.19	65.35	
7	M	5.70	82.71	5.70	82.78	30
	M±SD	6.30	79.28	6.30	79.32	
	M±2SD	6.89	75.86	6.89	75.86	
8	M	5.52	83.33	5.52	83.73	31
	M±SD	6.09	79.88	6.09	80.10	
	M±2SD	6.66	76.42	6.66	76.47	
9	M	5.39	84.24	5.21	84.75	33
	M±SD	5.95	81.11	5.76	81.89	
	M±2SD	6.51	77.97	6.30	79.03	
10	M	5.30	84.65	5.20	85.35	30
	M±SD	5.90	81.55	5.75	82.42	
	M±2SD	6.49	78.44	6.30	79.48	
11	M	5.10	85.56	5.17	85.45	30
	M±SD	5.76	82.33	5.76	82.39	
	M±2SD	6.42	79.11	6.35	79.32	

Table 5: Frequency Pattern results for children native speakers of European Portuguese aged 5 to 12 years: Percentage of correct answers for each age group for both ears and the number of segments in the pattern are presented. In addition to the average value, Average-1SD and Average-2SD are shown.

Age (years)		**Right Ear** 3 seg.	4 seg.	Total	**Left Ear** 3 seg.	4 seg.	Total	N
5	M	20.94	3.51	8.64	21.88	3.64	9.08	32
	M−SD	0	0	0	0	0	0	
	M−2SD	0	0	0	0	0	0	
6	M	54.31	27.03	35.47	54.66	27.04	35.75	31
	M−SD	28.87	2.63	12.67	28.49	3	13.56	
	M−2SD	3.44	0	0	2.32	0	0	
7	M	73.00	55.55	61.29	73.33	57.36	62.64	30
	M−SD	52.13	31.4	39.7	51.78	33.7	41.38	
	M−2SD	31.26	7.24	18.11	30.23	10.03	20.11	
8	M	83.55	66.07	71.25	85.48	67.01	72.58	31
	M−SD	68.82	46.1	53.71	68.19	46.48	54.03	
	M−2SD	54.09	26.13	36.16	50.9	25.94	35.49	
9	M	88.29	74.83	78.78	87.69	74.45	78.33	33
	M−SD	76.43	58.85	65.07	74.92	58.98	64.86	
	M−2SD	64.58	42.88	51.36	62.15	43.51	51.38	
10	M	91.33	81.39	84.24	91.33	80.83	83.75	30
	M−SD	79.09	69.77	74.29	78.27	70.13	74.63	
	M−2SD	66.85	58.15	64.35	65.21	59.43	65.52	
11	M	94.12	85.65	88.04	93.79	86.06	88.14	30
	M−SD	84.15	73.90	77.60	83.85	73.98	76.90	
	M−2SD	74.17	62.14	67.15	73.91	61.89	65.67	

Table 6: Duration Pattern results for children native speakers of European Portuguese aged 5 to 12 years: Percentage of correct answers for each age group for both ears and the number of segments in the pattern are presented. In addition to the average value, Average-1SD and Average-2SD are shown.

Age		**Right Ear**			**Left Ear**			
(years)		3 seg.	4 seg.	Total	3 seg.	4 seg.	Total	N
5	M	14.77	3.67	6.89	15.62	3.53	7.08	32
	M−SD	0	0	0	0	0	0	
	M−2SD	0	0	0	0	0	0	
6	M	44.06	19.31	27.12	44.20	20.14	27.72	31
	M−SD	14.44	0	5.39	14.06	0	5.93	
	M−2SD	0	0	0	0	0	0	
7	M	61.81	42.86	49.70	63.36	42.06	49.70	30
	M−SD	40.84	20.28	29.23	43.00	19.98	29.62	
	M−2SD	19.88	0.00	8.77	22.65	0.00	9.54	
8	M	74.63	56.50	62.04	74.66	57.73	62.90	31
	M−SD	60.55	40.73	47.51	60.52	42.71	49.19	
	M−2SD	46.48	24.96	32.98	46.38	27.69	35.49	
9	M	79.50	63.16	68.29	78.67	64.47	68.91	33
	M−SD	65.71	48.33	55.36	65.34	49.32	56.04	
	M−2SD	51.92	33.51	42.42	52.01	34.18	43.16	
10	M	85.30	71.86	77.15	85.90	71.14	76.87	30
	M−SD	74.58	57.78	66.51	73.65	57.21	65.39	
	M−2SD	63.86	43.70	55.87	61.40	43.28	53.91	
11	M	83.33	70.08	74.51	81.21	67.91	72.35	30
	M−SD	68.03	57.35	63.09	64.68	53.34	59.02	
	M−2SD	52.72	44.62	51.68	48.15	38.76	45.69	
